# Audio-digital recordings for surveillance in clinical trials of major depressive disorder

**DOI:** 10.1016/j.conctc.2019.100317

**Published:** 2019-01-08

**Authors:** Steven D. Targum, Christopher J. Catania

**Affiliations:** Bracket Global, 2 Oliver Street, Suite 1003, Boston, MA, 02109, USA

**Keywords:** Audio-digital recordings, Surveillance, MADRS, Remote ratings, Quality assurance

## Abstract

Ratings surveillance is used in clinical trials to assure ratings reliability of site-based scores. One surveillance method employs audio-digital recordings of site-based clinician interviews to obtain remote, site-independent scores for assessment of paired scoring concordance and interview quality. We examined the utility of this surveillance strategy using paired site-independent scores derived from recorded site-based Montgomery-Asberg depression rating scale (MADRS) interviews obtained from patients with major depressive disorder (MDD) participating in 5 clinical trials.

High correlations were noted between the 3736 paired site-based and site-independent scores across all visits. Some rater “outliers” were identified whose ratings performance improved following remediation. In 3 studies with available outcome data, the blinded remote ratings yielded a high predictive value (91.2%) for replicating treatment response rates.

The magnitude of the total MADRS scores affected the directionality of paired scoring deviations in each of the 5 studies. Across all visits, site-based raters scored the more severe MADRS scores (≥30) higher than site-independent raters and the less severe MADRS scores (<20) lower than site-independent raters. Individual MADRS items were similarly affected by the directionality of symptom severity.

This analysis affirms the utility of audio-digital recording of site-based interviews as a surveillance strategy for quality assurance (monitoring and remediation). In addition, the high predictive value of blinded remote ratings to replicate site-based treatment outcomes may be useful to affirm primary site-based results when there is a potential of functional unblinding. The use of remote ratings as a primary measure beyond its utility for quality assurance needs further exploration.

## 1 Introduction

The scores derived from clinical rating instruments administered during psychiatric clinical trials determine the efficacy of potential new drug candidates. Inter-rater scoring reliability can affect the power of the clinical trial to achieve signal detection [1]. Given its importance, elaborate rater training and certification programs have been designed to instruct, standardize, and subsequently demonstrate inter-rater reliability for each of the commonly used ratings instruments [2,3,5]. Nonetheless, a broad range of total scores often emerges when well-trained raters assess the same subject with an acute affective or psychotic disorder [3,6]. These inter-rater differences may be due to clinical judgement, a difficult subject, a lack of precision in the application of ratings conventions, or simply a lack of time given to adequately complete the instrument. Ratings inaccuracy (e.g. score inflation) due to misplaced site-based rater or subject motivations is also a form of deceptive practice that has been noted in clinical research [7].

Ratings precision during clinical trials presumes that raters will conduct complete interviews in a consistent manner at each study visit regardless of time pressures or other exigencies. In an analysis of 63 published papers, it was noted that few reports examine or describe the reliability of the ratings that were conducted during the study [8]. The use of remote, site-independent (centralized) raters in lieu of site-based raters has been suggested as an alternative method to optimize ratings in a clinical trial [4,[9], [10], [11], [12], [13], [14]]. Alternatively, site-independent review and scoring of site-based ratings has also been introduced as a quality assurance, surveillance strategy to monitor and assure ratings precision by site-based raters during a clinical trial [[15], [16], [17], [18]]. This strategy employs audio-digital recording and scoring of site-based interviews to obtain “paired” scores based upon the same interview.

In this report, we examined the utility of the audio-digital recording surveillance strategy from paired scoring data accumulated from 5 distinct clinical trials of major depressive disorder (MDD) that included 3736 site-based Montgomery-Asberg depression rating scale (MADRS) interviews [19].

## 2 Material and methods

Data for this ratings reliability analysis was obtained from 5 phase II or III clinical trials conducted between 2009 and 2017 as part of vendor grants awarded to Clintara LLC (or Bracket LLC) to conduct quality assurance/surveillance programs for ratings precision. The analysis was limited to double-blind, placebo-controlled trials that included patients with major depressive disorder (MDD) that used the MADRS as the primary efficacy measure and had obtained paired (“dual”) site-independent scores based upon audio-digital recordings of site-based interviews [16,17]. All enrolled subjects met DSM-IV criteria for MDD [20,21].

The 5 selected studies were registered in Clinicaltrials.gov as: NCT 01421134, 01500200, 02158533, 01912196, and 00739908. The work described in each study was carried out in accordance with The Code of Ethics of the World Medical Association (Declaration of Helsinki) for experiments involving humans. All patients provided written informed consent approved by an independent review board prior to participation in the studies.

All site-based and site-independent raters participated in comprehensive rater training and certification programs for each study that included didactic presentations, observation of expert MADRS video interviews, and demonstration of MADRS scoring competency via inter-rater reliability (IRR) assessments of the MADRS video interviews. Site-based raters were also required to demonstrate interviewing competency skills via mock MADRS interviews using a structured interview guide for the MADRS [22,23]. Across the 5 selected studies, paired MADRS scores were obtained from 397 certified site-based raters and 42 site-independent raters. Some of the raters participated in more than one study.

As part of this program, the site-based raters were trained to conduct the MADRS interviews using an audio-digital recording pen. The pens simultaneously audio-recorded the MADRS interview and digitally copied accompanying written notes that were captured on specially manufactured source books. The recorded interviews were electronically forwarded to Clintara LLC (Boston, MA) for random assignment to the site-independent raters who were blinded to the study visit, trial site, and site-based rater's scores. The site-independent raters generated their own paired (“dual”) scores by listening to the audio recording and reading the site-based rater's accompanying digital notes that did not contain scores.

The merged data from the 5 studies were examined to assess overall inter-rater reliability (IRR) and scoring concordance or deviations between the individual paired ratings. Scoring deviations were defined as the difference between the site-based score minus the site-independent score. Positive scoring deviations indicate that the site-based score was higher than the paired site-independent score. In addition, we examined the effect of the total MADRS score severity, study visit, and interview length on paired scoring deviations.

The total sample was divided into five sub-groups based upon total site-based MADRS scores at any study visit (≥40, 30–39, 20–29, 10–19, and 0–9) and four other sub-groups based upon the study visit itself (screen, baseline, post-randomization, and endpoint).

Statistical analyses used Students’ *t*-test and intra-class correlation (ICC) as appropriate to compare the site-based and site-independent scores relative to total and individual item MADRS scoring and timed interview length. The significance level was set at 5% for all tests in this analysis.

## 3 Results

There were 3736 MADRS “paired” scores available for site-based and site-independent ratings analyses.

### 3.1 Comparison of paired total MADRS scores

**Fig. 1:**
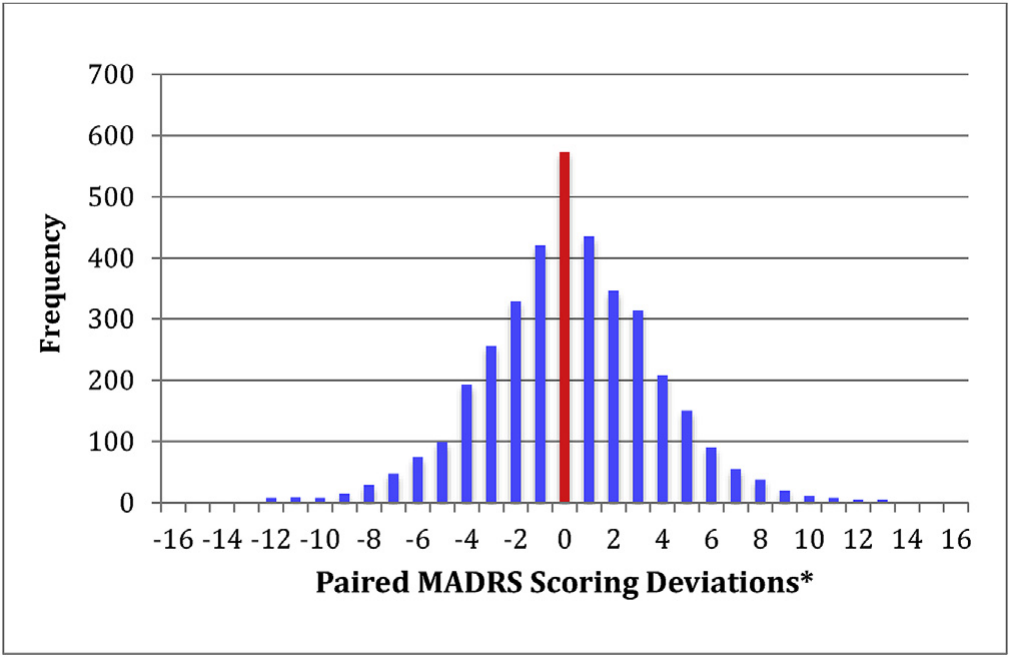
Distribution of paired MADRS scoring deviations (n = 3736). * Positive mean scoring deviations indicate that total site-based MADRS scores are higher than paired site-independent scores whereas negative deviations indicate that site-independent MADRS scores are higher than paired site-based scores.

As shown in Table 1 and Fig. 1, the paired total MADRS scores obtained at the screen, baseline, post-randomization and endpoint visits yielded high intra-class correlations (ICC) with minimal discordance.

**Table 1:** Paired site-based and site-independent MADRS scores across 5 studies.

Subject-visit	n	Mean SITE-based MADRS	Mean Independent MADRS	ICC	Mean Scoring Deviations	Discordant >6 points
ALL VISITS	3736	25.0 ± 10.8	24.8 ± 10.5	0.947	0.22	249 (6.7%)

SCREEN visit	296	31.9 ± 5.0	31.0 ± 5.5	0.749	0.96*	34 (11.5%)
BASELINE visit	1108	31.1 ± 6.1	30.8 ± 6.2	0.833	0.22	75 (6.8%)
Post-Randomization	2049	21.6 ± 11.2	21.4 ± 10.9	0.952	0.17	128 (6.3%)
Endpoint visit	283	19.0 ± 12.1	19.2 ± 11.5	0.966	−0.20	12 (4.2%)

*Site-based MADRS vs. site-independent MADRS scores at the screen visit: t = 2.22; df = 590; p = 0.027.

The site-based MADRS scores were significantly higher than site-independent scores at the screen visit (t = 2.22; df = 590; p = 0.027), but not significantly higher or lower at any other study visits.

There were 249 paired interviews (6.7%) with total scoring deviations >6 points in either direction. The surveillance strategy identified site-based raters who were frequent “outliers” and provided telephone ratings remediation. The basis for most paired ratings discrepancies was usually a failure to apply ratings conventions or interviews that were simply too short to conduct a comprehensive assessment. Subsequent review of site-based rater performance following telephone remediation revealed greater paired scoring concordance in almost every case. In 3 instances, the raters were removed from the study because they were not remediable.

### 3.2 Effect of total MADRS score on paired scoring deviations

**Fig. 2:**
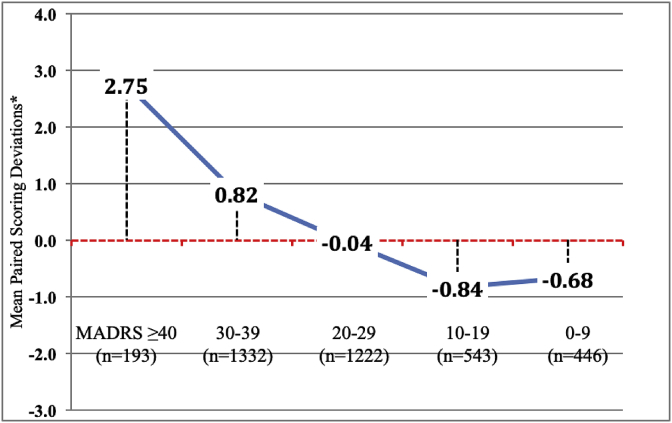
Effect of Total MADRS scores on Paired Scoring Deviations (all subjects). * Positive mean scoring deviations indicate that total site-based MADRS scores are higher than paired site-independent scores whereas negative deviations indicate that site-independent MADRS scores are higher than paired site-based scores.

**Fig. 3:**
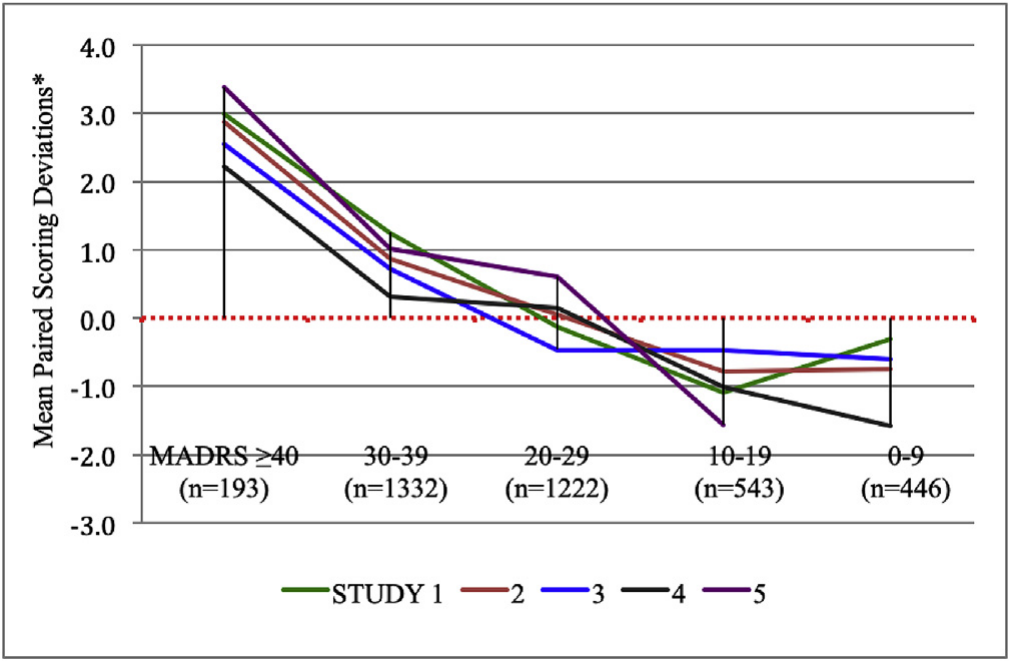
Effect of Total MADRS score on Paired Scoring Deviations across 5 Studies. * Positive mean scoring deviations (above 0.0) indicate that site-based MADRS scores are higher than paired site-independent scores whereas negative deviations indicate that site-independent MADRS scores are higher than paired site-based MADRS scores.

The magnitude of the total site-based MADRS score affected the paired scoring deviations. As shown in Fig. 2, Fig. 3, high or low total MADRS scores determined the directionality of the scoring deviations in each of the 5 studies examined. Site-based raters tended to score the higher MADRS scores (≥30) higher than the paired site-independent scores and the lower MADRS scores (<20) lower than paired site-independent scores (Table 2).

**Table 2:** Paired site-based and site-independent individual MADRS item comparisons.

a. Individual MADRS item scoring deviations relative to total score severity											
	Item 1	Item 2	Item 3	Item 4	Item 5	Item 6	Item 7	Item 8	Item 9	Item 10	Total Score
All Subjects	−0.07	0.06	−0.05	0.06	0.02	0.03	0.10	−0.02	0.00	0.03	0.22
ICC	0.855	0.884	0.861	0.917	0.875	0.853	0.840	0.868	0.882	0.929	0.947
MADRS ≥40	0.34	0.39	0.26	0.26	0.39	0.32	0.42	0.22	0.17	0.15	2.75
30–39	0.02	0.12	0.04	0.15	0.10	0.11	0.20	0.05	0.07	0.07	0.82
20–29	−0.08	0.01	−0.09	0.09	−0.01	0.02	0.15	−0.06	−0.01	0.02	−0.04
10–19	−0.22	−0.02	−0.12	−0.09	−0.08	−0.05	−0.11	−0.08	−0.07	−0.01	−0.84
0–9	−0.17	0.03	−0.13	−0.04	−0.03	−0.07	−0.07	−0.04	−0.08	−0.01	−0.68
MADRS Items: Item 1.: Reported sadness; Item 2: Apparent sadness; Item 3: Inner tension; Item 4: Reduced sleep; Item 5: Reduced appetite; Item 6: Concentration difficulties; Item 7: Lassitude; Item 8: Inability to feel; Item 9: Pessimistic thoughts; Item 10: Suicidal thoughts											

b. Significant T tests after bonferroni correction for individual MADRS items											
	Item 1	Item 2	Item 3	Item 4	Item 5	Item 6	Item 7	Item 8	Item 9	Item 10	Total Score
All Subjects	ns*	ns	ns	ns	ns	ns	ns	ns	ns	ns	ns
MADRS ≥40	0.004	0.003	ns	ns	ns	0.002	<0.0001	0.140	ns	ns	<0.0001
30–39	ns	0.010	ns	0.100	ns	ns	<0.0001	ns	ns	ns	<0.0001
20–29	ns	ns	ns	ns	ns	ns	ns	ns	ns	ns	ns
10–19	0.050	ns	ns	ns	ns	ns	ns	ns	ns	ns	0.004
0–9	0.090	ns	0.100	ns	ns	ns	ns	ns	ns	ns	0.040

* ns = T test was not significant after bonferroni correction.

There were 136 paired MADRS scores (3.6% of all scores) with >6 points positive scoring deviations (indicating that the site-based total scores were higher than the site-independent scores) and 113 paired scores (3.0% of all paired scores) with negative deviations. As noted above, the magnitude of the total MADRS scores affected the directionality of the scoring deviations. Thus, the mean total MADRS score was 31.8 ± 8.1 (SD) amongst the 136 paired scores with positive deviations and only 21.7 ± 8.0 amongst the 113 paired scores with negative scoring deviations (t = 9.83; df = 247; p < 0.0001).

**Fig. 4:**
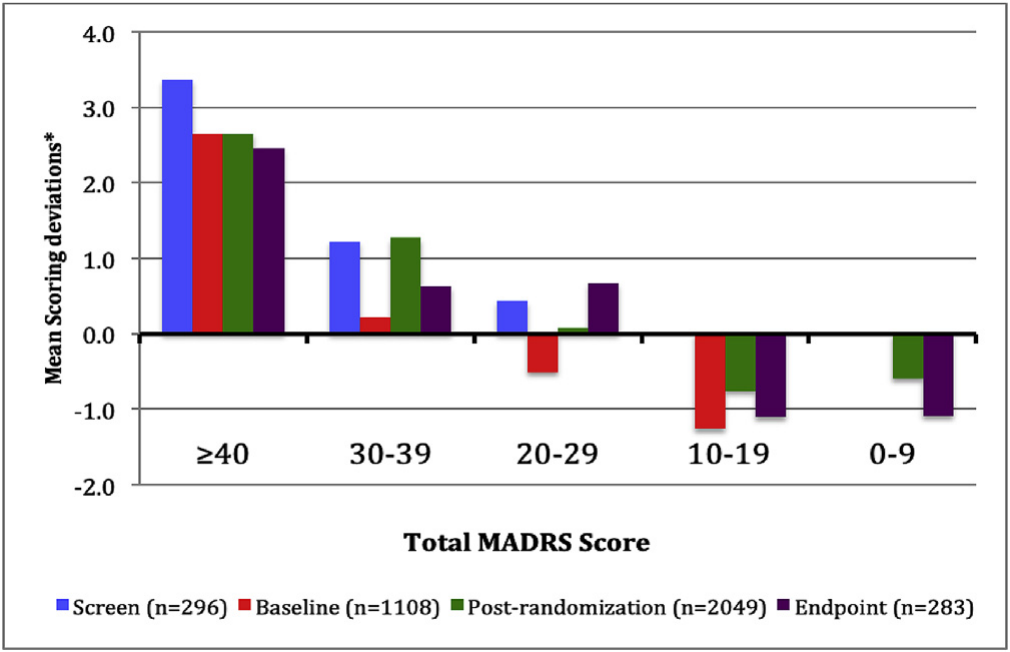
Mean Paired MADRS Scoring Deviations relative to Total Score and Study Visit. * Positive mean scoring deviations indicate that site-based MADRS scores are higher than paired site-independent scores whereas negative deviations indicate that site-independent MADRS scores are higher than paired site-based MADRS scores.

The magnitude of total MADRS scores affected mean paired scoring deviations across all visits. Higher total MADRS scores yielded more positive paired scoring deviations across all visits whereas lower scores tended to yield more negative scoring deviations regardless of the study visit (Fig. 4).

### 3.3 Paired scoring comparison of individual MADRS items

The paired scores yielded a high ICC for each MADRS item with minimal scoring disagreement. However, the directionality of the paired scoring deviations of each of the 10 individual MADRS items was affected by the magnitude of the endorsed symptom severity. Table 2 details the mean paired scoring deviations observed for each of the 10 MADRS items and the total MADRS score. For total scores ≥40 the site-based scores were significantly higher than site-independent scores for items 1, 2, 6, and 7. Alternatively, for total MADRS scores <20 the site-based scores were lower than site-independent scores for item 1 (reported sadness).

Individual item paired scoring differences were usually within one point of each other in either direction. For instance, the scoring difference was within one point in 96.4% of MADRS interviews for item 1 (reported sadness) of which 61.1% were exactly the same.

Accurate scoring of MADRS item 2 (observed sadness) by listening to an audio recording is obviously limited by virtue of no visual observation. The structured interview guides used in these studies provided specific queries that generated useful information to facilitate scoring [22,23]. For instance: Do you think you have looked sad or depressed to other people? Did anyone say you looked sad or down? Has it been hard for you to laugh or smile in the past week? This information is usually sufficient to obtain concordant paired scores. In the current analysis, the ICC between the paired site-based and independent scores was 0.884 (Table 2). There was a close scoring correspondence between items 1 (reported sadness) and 2. The ICC between items 1 and 2 was 0.876 and 89.9% of item 1–2 scores were within one point of each other.

We compared the use of the full MADRS scoring range (0–6) by site-based and site-independent raters. Site-independent raters used scores of 6 (most extreme) or 0 (no symptom endorsement) as often as site-based raters. For instance, site-based raters endorsed a score of 6 in 1.7% of interviews for MADRS item 1 versus 1.5% by site-independent raters and site-based raters endorsed a score of 0 in 13.3% of interviews versus 13.3% by site-independent raters.

### 3.4 Predictive value of independent MADRS paired ratings

Although most programs recorded 100% of MADRS interviews throughout a study, only a small percentage of MADRS scores were actually scored by site-independent raters at the endpoint of each study. Consequently, paired MADRS scores for both the baseline and study endpoint visits for the same subject were available from only a small sub-group of study subjects. Merging data from 3 of the studies in this analysis, 196 of the 215 site-independent score pairs (91.2%) correctly matched the response/non-response outcomes of the site-based raters with little variation between the 3 studies (88.7%, 100%, and 92.9%).

### 3.5 Effect of interview length on paired scoring deviations

There were 1235 paired MADRS scores with reliable interview length data in this merged data sample. Recorded MADRS interview lengths range from 3:30 to 62:26 min (mean interview length = 17:37 ± 8:16 min). The mean interview length for the screen and baseline MADRS interviews was 21:17 ± 8:38 min and were significantly longer than the post-randomization and endpoint interviews (15:42 ± 7:22) due to the extent of symptom endorsement and the higher total MADRS scores at the early visits (t = 11.93; p < 0.0001). The truncated scoring range at screen and baseline (due to the specified minimum eligibility criteria for each study) yielded a modest correlation between interview length and the site-based total MADRS score (r = 0.113; df = 423; p = 0.02) in contrast to the post-randomization correlation (r = 0.416; df = 808; p < 0.0001) that included a broader MADRS scoring range from 0 to 52.

In a previous report, we noted that site-based MADRS interviews conducted in ≤12 min yielded significantly greater paired scoring deviations than longer interviews at the screen visit (Targum et al., in press). However, with one exception noted below, interview length was not associated with paired MADRS scoring deviations at the screen, baseline, or post-randomization visits in this larger analysis.

In this analysis, we found that total MADRS scores ≥40 were affected by interview length at any visit. Thus, 5 of 10 paired screen interviews with MADRS scores ≥40 and interview lengths ≤12 min had >6 point scoring deviations (50%) in contrast to 9 of 52 interviews (17.3%) that were longer regardless of the study visit (Fisher exact test = 0.038). These ratings “outliers” were identified and remediated.

## 4 Discussion

We examined the utility of audio-digital recordings as a surveillance strategy for quality assurance of site-based interviews from merged data from 5 clinical studies of MDD. Site-independent scoring of the audio-digital recordings of 3736 site-based MADRS interviews yielded highly reliable paired scores (r = 0.947 for all interviews) with minimal scoring deviations. The high correlation found been site-based and remote scores based on audio recordings is consistent with the report of [4] who found similarly high correlations between site-based ratings and separately conducted remote telephone or video-based MADRS interviews. Paired scoring reliability was observed across the screen, baseline, post-randomization, and endpoint visits. Further, the paired MADRS scores yielded a high predictive value (91.2%) for treatment response in a sub-set of 215 subjects with paired baseline and endpoint data.

The above findings from 5 different MDD studies affirm the utility of audio-digital recording of MADRS interviews as a quality assurance method to optimize site-based ratings precision. Of course, ratings reliability is contingent upon competent site-based interviews. This surveillance strategy reinforces competent ratings performance because raters are aware that their recorded interviews are subject to independent review and monitoring. Nonetheless, some raters conducted shorter, incomplete interviews or failed to correctly apply ratings conventions. We have found that telephone remediation of rater “outliers” who exceeded pre-specified paired concordance ranges contributes to improved ratings performance on subsequent MADRS interviews in most instances.

Most of the 3736 MADRS interviews were complete and judged to be of good quality by the independent raters. The good interview quality may have been reinforced by the rater's awareness of the audio-digital recording surveillance strategy. Further, the actual interview length did not affect paired scoring concordance in most of 1235 timed MADRS interviews with the exception of short interviews (≤12 min) conducted in patients with MADRS scores ≥ 40 at any visit.

The mean total site-based MADRS scores were significantly higher than the paired site-independent scores at the screen visit (p = 0.027). Although this significant paired scoring difference might reflect some site-based score inflation to meet study eligibility criteria, the difference was affected by the magnitude (severity) of the total MADRS score at the screen visit as well. In fact, symptom severity based on the magnitude of the total MADRS score was the primary driver of paired scoring concordance or deviations across all visits. High total site-based MADRS scores (≥40) generated significantly greater positive scoring deviations (site-based scores > site-independent scores) regardless of the study visit (as reflected in Fig. 4). Site-based scores for individual items 1 and 2 (reported and observed sadness), item 6 (concentration difficulties), and item 7 (lassitude) were significantly greater than site-independent scores for the highest total MADRS scores. Alternatively, the lower total site-based MADRS scores yielded significantly more negative scoring deviations, particularly for MADRS item 1 in which site-based scores were lower than site-independent scores.

Why is there a paired scoring difference between site-based and site-independent raters at the upper and lower ranges of MADRS symptom severity? It is not because site-independent raters were reluctant to use the full 0–6 scoring range of each MADRS item. In fact, the individual item scoring disagreements were usually within one point of each other in either direction. Table 2 demonstrates that the slight paired scoring differences occurred with every MADRS item based upon the level of symptom severity. Further, the slight paired scoring difference is not due to the site-based raters awareness of the visit trajectory from screen to endpoint because they still scored patients with greater symptom severity slightly higher than remote raters during post-randomization and endpoint visits. The observed paired scoring differences may simply be due to a non-quantifiable clinical nuance that is possible during a live interview that cannot be matched by simply listening to an audio recording of the same interview.

However, it is noteworthy that live remote interviews have also been shown to generate scoring deviations between site-based and remotely scored interviews in some clinical studies. There have been few published reports that directly compared live site-based interviews with live remote ratings through the course of an entire clinical trial. In one recent clinical study that used live, remote telephone ratings of the Hamilton rating scale for anxiety (Ham-A) in patients with generalized anxiety disorder (GAD), the centralized Ham-A scores were lower at the baseline visit and higher at the endpoint than the site-based scores [13]. In another study that used video-based centralized ratings of the Inventory of Depressive Symptomatology (IDSc30) in acute MDD study, the centralized scores were also lower at baseline and higher at the endpoint than the site-based scores [24]. The findings from these two studies using different remote ascertainment methods to obtain site-independent scores are consistent with the findings in the current analysis of audio-digital recordings for remote scoring. Therefore, our findings may have broader implications for all methods of remote (centralized) ratings. Clearly, these observations are based on just a few studies and further exploration with other data sets are needed.

In summary, the current analysis of 3736 paired MADRS scores from 5 clinical studies affirms the utility of audio recording of site-based interviews as a surveillance strategy for site-independent quality assurance (monitoring and remediation). This method can effectively allay concerns about deceptive ratings practices [7]. In addition, the high predictive value of blinded remote ratings to replicate site-based treatment outcomes may be useful to affirm primary site-based results when there is a potential of functional unblinding. The use of remote ratings as a primary measure beyond its utility for quality assurance needs further exploration.

## Contributors

Dr. Targum participated in the design, implementation, and analysis of the original studies and conceived, analyzed, and wrote the current analysis reported in this manuscript. Mr. Catania assisted with the collection, collation, and analysis of the data and assisted with the preparation of the final manuscript.

## Conflicts of interest

Dr. Targum is currently Scientific Director at Bracket Global LLC., and was previously at Clintara LLC during which time vendor grants were received to conduct quality assurance programs for the 5 specific studies in this analysis from Alkermes Inc., CeNeRx, Methylation Sciences Inc., and Sunovion Pharmaceuticals. Dr. Targum study has received no compensation or input from these sponsors for the analysis or preparation of this specific manuscript. He has also received consultation fees or vendor grants from Acadia Pharmaceuticals, AZ Therapies, Brain Cells Inc., Forum Pharmaceuticals, Functional Neuromodulation Inc., Intracellular Therapies, Inc., Johnson and Johnson PRD, Karuna Pharmaceuticals, Navitor Pharmaceuticals, Neurim Pharmaceuticals, Prana Biotechnology Ltd., Pfizer Inc., and Resilience Therapeutics.

Christopher J. Catania is an employee of Bracket Global and has no other disclosures.

## Role of the funding source

Partial support for this study came from vendor grants to conduct quality assurance programs from Alkermes Inc., CeNeRx, Methylation Sciences Inc., and Sunovion Pharmaceuticals with additional support for the analyses from Bracket Global LLC (Wayne, PA). However, neither Bracket Global LLC nor any sponsor had any role in the analysis and/or interpretation of the data, the writing of this report, or the decision to submit the manuscript in its current form.
